# A Case of Critical Digit Ischemia in a Patient With Suspected Seronegative Scleroderma Diagnosed via Nailfold Capillaroscopy

**DOI:** 10.1155/crrh/7668313

**Published:** 2026-08-19

**Authors:** Matthew Kornas, Charis Wang, Sehreen Mumtaz, Adrian G. Dumitrascu, Florentina Berianu

**Affiliations:** ^1^ Department of Internal Medicine, Division of Hospital Internal Medicine, Mayo Clinic, Jacksonville, Florida, USA, mayo.edu; ^2^ Alix School of Medicine, Mayo Clinic, Phoenix, Arizona, USA, mayo.edu; ^3^ Department of Internal Medicine, Division of Rheumatology, Mayo Clinic, Jacksonville, Florida, USA, mayo.edu

## Abstract

Systemic sclerosis (scleroderma) can manifest with advanced symptoms of ischemic vasculopathy, including digital ulcer development. These ulcerations can be disabling and detrimental to a patient’s quality of life, so prompt diagnosis, differentiation from other vascular etiologies, and treatment are important to help salvage this tissue. However, diagnosis of scleroderma can be challenging in the setting of seronegative disease. Nailfold capillaroscopy is a vital tool that can assist with diagnosis in seronegative cases. Scleroderma pattern findings on nailfold capillaroscopy include dilated or giant capillaries, capillary hemorrhages, and dropouts. Pharmacologic and surgical modalities can effectively treat vascular complications from scleroderma. Vasodilation therapy with prostacyclin analogs, calcium channel blockers, or angiotensin II receptor blockers can improve ischemic tissue perfusion. Surgical sympathectomy should be offered to treat ulcerative tissue and improve related pain control. We present a case of seronegative scleroderma in a patient with digital ischemic ulcerations that was expediently diagnosed and treated with assistance of nailfold capillaroscopy.

## 1. Introduction

Systemic sclerosis (scleroderma) is an autoimmune connective tissue disease that affects multiple organ systems via fibrotic changes and vascular injury. Such pathophysiology includes several hallmark cutaneous findings, including Raynaud’s phenomenon, skin fibrosis, and digital ulcerations or gangrene [1, 2]. The latter findings can occur due to microvascular dysfunction in the setting of reduced peripheral perfusion in patients afflicted with this disease. Use of Laser Doppler perfusion imaging to assess perfusion in the hands of scleroderma patients after cold stimulation noted significantly reduced baseline mean perfusion and skin flow recovery compared to healthy controls [3]. Additional research has found that skin blood perfusion measured by laser speckle contrast analysis had a significant inverse correlation with dermal thickness measured by ultrasound at the dorsum of the middle phalanx of the third finger [4]. As such, use of these imaging modalities together was proposed as non‐invasive techniques to objectively assess disease response to treatment [4].

Scleroderma is frequently associated with the presence of autoantibodies. Previous literature that examined a western Indian population with scleroderma revealed that 98% of patients exhibited a positive antinuclear antibody (ANA) and 81% had a positive antitopoisomerase (anti‐Scl‐70) antibody [5]. Rheumatologic serology is often utilized to help diagnose scleroderma, including in the 2013 American College of Rheumatology/European League Against Rheumatism (ACR/EULAR) 2013 guidelines [6]. Cases of scleroderma that are seronegative (or lack positive autoantibody results) can therefore be more challenging to promptly diagnose, which can delay treatment of disease‐related complications.

Critical digit ischemia is a rare but debilitating late complication of scleroderma. It is characterized by impaired tissue perfusion that manifests severe pain at rest and visible distal tissue ulceration, necrosis, or gangrene [7, 8]. Findings of necrotic or gangrenous changes represent an advanced end of the spectrum of digital vasculopathy [7]. Digital ulcerations are more common among these patients and represent ischemic injury that is reversible if prompt treatment is initiated [8]. Evaluation for other vascular diseases or etiologies is also important to exclude concomitant diseases that require individualized treatment. Medical therapies include vasoactive agents such as calcium channel blockers, angiotensin‐converting enzyme inhibitors, and prostanoids [8]. Surgical interventions may be required in select patients and vary based on disease progression, including debridement of necrotic tissue and sympathectomy of ulcerated but viable tissue [8].

In this case, we present a patient with signs and symptoms of critical digit ischemia of multiple fingertips. Prior vascular and cardiology workup did not elucidate a distinct vascular disease—and serology evaluation at our facility was negative. We performed nailfold capillaroscopy and found features distinctive for scleroderma, supporting a diagnosis of seronegative scleroderma given negative autoantibody testing. She was initiated on dual pharmacologic and surgical therapy with clinical improvement as noted in the following case presentation.

## 2. Case Presentation

A 47‐year‐old female with history of tobacco use initially presented to outside medical providers in late 2024 with a ten‐month history of episodic fingertip discoloration, pain, and numbness. Vascular surgery, cardiology, and rheumatology providers evaluated her, with an extensive workup unable to reveal a definitive etiology for her symptoms. This included a CT angiogram of the upper extremities, which showed adequate perfusion above the elbows with diminished arterial flow in the ulnar arteries and vascular arches of the hands. She was diagnosed with Raynaud’s phenomenon and underwent medical therapy with sildenafil, calcium channel blockers, apixaban, and topical nitroglycerin at varying time intervals. These therapies failed to improve her symptoms, and she subsequently presented to our facility in February 2025 for a second opinion via expedited inpatient workup.

**FIGURE 1:**
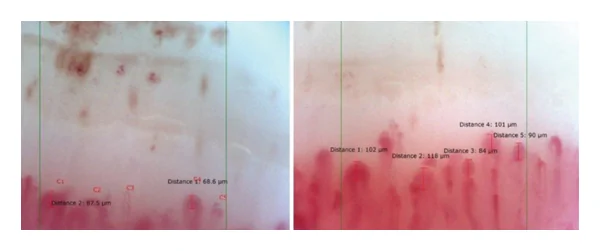
Nailfold capillaroscopy images of the bilateral index fingers with findings representative of an active scleroderma pattern. Vertical green lines denote the borders of a 1 mm area. Left image demonstrates capillaries (C1–C5) with a low capillary density of 5 capillaries/mm (normal > 7 capillaries/mm). Notable microhemorrhages are present in the upper portion of the image. Right image shows multiple giant capillaries measuring up to 118 microns in apical diameter.

Examination showed dactylitis with ischemic ulcerations and eschars of the second and third right fingertips plus skin breakdown indicative of early second and fifth left fingertip ulcerations. Rheumatic serologic workup was unremarkable with negative centromere, anti‐Ro (SS‐A), anti‐La (SS‐B), Smith, ribonucleoprotein (RNP), topoisomerase I (Scl‐70), and histidyl‐tRNA synthetase (Jo‐1) antibodies plus normal ANA, complement, and cryoglobulin levels. Nailfold video capillaroscopy of multiple digits demonstrated an active scleroderma pattern with multiple giant capillaries and capillary microhemorrhages (Figure 1). She was subsequently diagnosed with seronegative scleroderma complicated by secondary Raynaud’s phenomenon and dry gangrene of multiple digits.

Our team recommended both medical and surgical management of her disease. Hydroxychloroquine 200 mg, prednisone 10 mg, amlodipine 2.5 mg, and aspirin 81 mg were initiated. We advised her to utilize a nitroglycerin patch upon the proximal affected fingers to enhance vasodilation. Due to the severity of her symptoms, progressive ischemic changes, and lack of response to outside pharmacologic therapies, we recommended therapeutic surgical sympathectomy to provide more definitive relief. During her hospitalization, she was evaluated by orthopedic surgery and anesthesia with a trial chemical sympathectomy. This demonstrated increased vascular resistance distally in the right upper extremity on pre‐injection ultrasound followed by normal biphasic flow representing vessel relaxation after injection of ropivacaine. This indicated that a component of sympathetic tone was contributing to her symptoms, and she was therefore a candidate for surgical sympathectomy. The patient elected to trial medical management upon discharge with outpatient elective sympathectomy if symptoms did not improve.

Chemical sympathetic nerve block of the right upper extremity in the outpatient setting improved distal blood flow through the radial and ulnar arteries measured by Doppler exam. She underwent surgical sympathectomy of the right radial, ulnar, and common digital arteries in March 2025. She subsequently underwent surgical sympathectomy of the left radial, ulnar, and common digital arteries in April 2025. Examination in April 2025 after both procedures showed healed eschars of the right second and third fingertips along with improved pain and function. At the time of her last visit with her primary care provider in December 2025, the patient was completing a prednisone taper prescribed by her outside rheumatologist with physical exam documentation noting no evidence of color change, pallor, or wounds.

## 3. Discussion

This patient met criteria for the diagnosis of scleroderma with a score of 9 points using the 2013 ACR/EULAR criteria: puffy fingers (2 points), digital tip ulcers (2 points), abnormal nailfold capillaries (2 points), and Raynaud’s (3 points). Scleroderma pattern findings on nailfold capillaroscopy include dilated or giant capillaries caused by an angiogenic response to peripheral ischemia, capillary hemorrhages as an example of Raynaud’s phenomenon, and dropouts [1, 5]. Nailfold capillaroscopy can add objective information and insight for cases where the antibody profile may not be supportive for atypical presentations of scleroderma, such as in this seronegative case [1].

The patient’s differential diagnosis included other vasculitides such as thromboangiitis obliterans (Buerger’s disease) along with nonrheumatic etiologies, including peripheral arterial disease. Nailfold capillary changes can occur in Buerger’s disease, and it is notable that the typical triphasic discoloration of the digits was lacking in this case. While Buerger’s disease was considered, the presence of microvascular changes of the scleroderma pattern was deemed to support the diagnosis of scleroderma. The pathophysiology of this disease can result in distinct capillaroscopic patterns with capillary loss, with the presence of giant capillaries being highly pathognomonic for scleroderma. Buerger’s disease involves small and medium‐sized arteries and veins rather than capillaries, leading to relatively preserved nailfold capillaries. Using the Very Early Diagnosis of Systemic Sclerosis (VEDOSS) criteria, the combination of puffy fingers and a scleroderma pattern on nailfold video capillaroscopy supports the diagnosis of systemic sclerosis despite negative SSc‐specific autoantibodies [9]. In addition to the patient’s clinical and capillaroscopy findings, other autoimmune etiologies such as systemic lupus erythematosus, cryoglobulinemia, and mixed connective tissue disease were deemed less likely due to the patient’s negative serology results (including negative ANA and anti‐RNP antibodies along with normal cryoglobulin levels).

Peripheral arterial disease was also considered given the patient’s reported diminished arterial flow in the ulnar arteries and vascular arches of the hands on outside angiography evaluation. Her puffy fingers may have been caused by reactive edema related to digit ischemia as well. However, evaluation by an outside vascular surgeon deemed that the patient’s presentation was not consistent with peripheral vascular disease. While tobacco use was present as a risk factor, her medical history also lacked diagnoses of hypertension, diabetes, and hyperlipidemia that are frequently present with this diagnosis.

While characteristic pattern findings were visualized on nailfold capillaroscopy in this case, its use does have potential limitations. Physiologic factors can impact image quality via multiple mechanisms, including that tobacco and alcohol use can alter capillary diameter and circulation [10]. In addition, capillaroscopy findings can be limited in patients with thick and dark skin due to the proportional absorption of visible light by melanin [10]. It is also important to note that human error can impact both the operator of the instrument and the interpretation of its images. Therefore, it is recommended that personnel with adequate training be tasked with performing this evaluation.

Cases of seronegative scleroderma remain rare, contributing to limited available literature to guide treatment. One study identified a total of 208 out of 3249 patients (6.4%) diagnosed with scleroderma but ANA‐negative [11]. Interestingly, these patients were found to have significantly less vasculopathic manifestations, including digital ulcers [11]. Treatment recommendations were therefore based on established guidelines for scleroderma care. The patient was initiated on hydroxychloroquine and prednisone for immunomodulatory therapy, with mycophenolate mofetil later added by her outpatient rheumatologist. The patient was also provided multimodal medical therapy for her vasculopathic symptoms (Raynaud’s phenomenon and digital ulcers), including amlodipine and nitroglycerin patch for vasodilation, aspirin for antiplatelet therapy, and atorvastatin. Prior literature has shown vasodilator therapy with prostacyclin analogs, calcium channel blockers, or angiotensin II receptor blockers (ARBs) as vital to improve perfusion to ischemic tissues [2, 7, 12]. Calcium channel blockers and phosphodiesterase type 5 inhibitors have particularly been identified as agents with a strong level of evidence for use in this context [13]. Anticoagulation or antiplatelet therapy may theoretically provide benefit in the setting of scleroderma‐induced platelet activation, but more research is required to better determine its benefits [7, 12]. Use of statin therapy or aspirin has a modest level of review in the literature but is more conditionally recommended [13].

Additionally, the patient subsequently underwent therapeutic sympathectomy for more definitive management of her symptomatic ischemic digital ulcers. A previous report summarized that surgical sympathectomy provides effective pain relief with resolution of digital ulcerations in most cases [14]. The patient underwent bilateral upper extremity sympathectomy with no apparent complications—with subsequent complete lesion healing and normal epithelium on the involved fingertips. Such a case re‐emphasizes the importance of surgical sympathectomy as a management option for patients with critical digit ischemia. In conclusion, a complete workup including the use of nailfold capillaroscopy for patients with peripheral ischemic vasculopathy, is paramount for a correct diagnosis and can guide an expedited treatment aiming to prevent hand structure and function loss.

## Author Contributions

Sehreen Mumtaz and Florentina Berianu were responsible for data collection and interpretation, article editing, and analysis. Matthew Kornas was responsible for data interpretation, article writing, and editing of the final manuscript. Charis Wang, Sehreen Mumtaz, Adrian G. Dumitrascu, and Florentina Berianu were responsible for reviewing and offering editorial feedback on the manuscript. Florentina Berianu supervised the project.

## Funding

No specific funding was provided for the medical care nor manuscript creation of this case report.

## Disclosure

All authors had the opportunity to review and approve the final manuscript before publication.

## Consent

Patient consent was obtained for the publication of this report. Nonessential patient identifying information has been withheld in this publication in accordance with ICMJE guidelines.

## Conflicts of Interest

The authors declare no conflicts of interest.

## Data Availability

The medical data for this case report is available upon reasonable request to the corresponding author.
